# There is no donor side specificity of fibula free flap for complex oromandibular reconstruction

**DOI:** 10.4103/0970-0358.73438

**Published:** 2010

**Authors:** Prabha S. Yadav, Quazi G. Ahmad, Vinay Kant Shankhdhar, G. I. Nambi

**Affiliations:** Plastic and Reconstructive Services, Department of Surgical Oncology, TATA Memorial Hospital, Parel, Mumbai - 400 012, India

**Keywords:** Complex oromandibular defect, oromandibular reconstruction, free fibula flap, donor side specificity

## Abstract

**Background:**

The objective of this study was to prove that there is no significance to the donor side (right or left) of the free fibula osteocutaneous flap (FFOCF) in the reconstruction of complex oromandibular defects (COMD) and proper flap planning, designing and tailoring are important in reconstructing different types of COMD after tumour-ablative surgery.

**Materials and Methods:**

Three hundred and eighty-six consecutive patients who where reconstructed with FFOCF for COMD from Jan 2005 to Dec 2009 over a period of 5 years were studied. Except in seven patients, all fibula flaps were harvested from the left leg as per convenience and to facilitate a simultaneous, two-team approach. Depending on the condition of the neck vessels, vascular anastomosis was performed on the right or the left side, irrespective of the side of the defect.

**Results:**

Complete flap survival was seen in 334 patients (86.52%). Superficial skin necrosis was seen in 20 patients, and was managed conservatively (5.18%). Partial flap loss was seen in 20 patients (5.18%). There were 39 re-explorations. Complete flap loss was seen in 12 patients (3.10%).

**Conclusion:**

We found no significance in terms of the results as far as the side of flap donor leg or primary defect were concerned. Flap tailoring in terms of meeting the tissue requirement and vessel orientation were rather more important.

## INTRODUCTION

The free fibula flap is being used most commonly for the reconstruction of complex oromandibular defects (COMD) owing to the advantages of its versatility.[1] According to the literature reports, the choice of donor side selection (ipsilateral or contralateral) in harvesting the free fibula flap depends on the side of the defect, need for skin cover, intraoral or extraoral soft tissue defect and availability of recipient blood vessels in the neck.[2,3] However, a 5-year experience from our centre proves that irrespective of the side of the defect and availability of recipient blood vessels, the free fibula flap harvested from any side (right leg or left leg) can be used to reconstruct the COMD without compromising the structural and functional outcomes.

## MATERIALS AND METHODS

Three hundred and eighty-six consecutive patients who where reconstructed with free fibula osteomyocutaneous flap (FFOCF) for COMD from Jan 2005 to Dec 2009 over a period of 5 years were studied. Except in seven patients, the remaining flaps were harvested from the left leg as per convenience to avoid crowding of the operative teams on one side and to facilitate the two-team approach with ease. Of these seven patients, one had poliomyelitis, two had previous surgeries for trauma and in the remaining four, the dorsalis pedis and the posterior tibial pulsations could not be assessed neither clinically nor with hand Doppler. To remove the technique-dependent bias, all the flaps were harvested by the standard anterior technique and to remove the operator-dependent bias, all the flaps were performed by different surgeons with varying levels of experience. Hand Doppler marking of the skin perforators supplying the lateral leg over the posterolateral septum was performed and skin paddle of varying sizes and the flexor hallucis longus muscle (FHL) was included in all the flaps. Depending on the condition of the neck vessels, the vascular anastomosis was performed on the right or the left side, irrespective of the side of the defect, and vein grafts were not used in any case. All patients underwent tracheostomy to facilitate post-op ventilation.

There were 270 males and 116 females. The age of the patients ranged from 8 to 73 years. The most common pathology was squamous cell carcinoma. There were 262 central segment defects and 31 right lateral segment defects and 93 left lateral segment defects. The facial artery was used for anastomosis in 316 patients, the superior thyroid in 62 patients and the transverse cervical artery in eight patients. The external jugular vein was used in 42, tributary of the internal jugular vein was used in 252 patients and both were used in 92 patients. The right side vessels were used for anastomosis in 147 patients and the left side vessels in 239 patients.

### The technique

All the flaps were harvested by standard anterior approach.[4] The flap donor site was covered with a split skin graft from the opposite thigh depending on the size of the skin paddle included. The maximum length of the fibula was harvested, leaving behind the proximal and distal 6 cm. The length of the skin paddle ranged from 15 to 30 cm and the width from 3 to 12 cm, depending on the requirement. The vertical extent of the skin paddle was confined within the levels of osteotomy and the horizontal extent was between the posterior midline of the leg to 1 cm lateral to the shin of the tibia. After the flap harvest, the oromandibular defect and the availability of the recipient vessels in the neck were assessed. After this, the osteotomies were planned from the distal to the proximal end of the fibula flap so that the excess bone could be discarded and the pedicle length could be increased. The peroneal surface of the fibula was used for the fixation of miniplates and screws. In all the flaps, the osteotomy and modelling of the neomandible was carried out after detaching the flap from the leg.

### The design

For anastomosis on the left side, when the flap was harvested from the left leg [Figures 1a and b], the FHL muscle comes to lie in the submental region and the skin paddle drapes the bone from outside the oral cavity as the septal attachment to the bone comes to lie at the lower border of the neomandible [Figures 2a and b]. For anastomosis on the right side, with a flap from the left leg, the FHL muscle comes to lie in the floor of the mouth and the skin paddle drapes the bone from inside the oral cavity as the septal attachment to the bone comes to lie at the upper border of the neomandible [Figures 3a and b]. Therefore, with this design, in a left side fibula flap with anastomosis on the ipsilateral side, the septum covers the peroneal surface of the bone with plate and screws from the outside and, for a left side fibula flap with anastomosis on the right side, the skin paddle covers the peroneal surface with plate and screws from inside. This was possible because of the flexibility of the postero-lateral septum. With this technique, we were able to anastomose on the opposite vessels even in the reconstruction of the lateral defects as the pedicle comes to lie more anteriorly. The same design and technique was used vice versa in the reconstruction of the COMD, with free fibula flap from the right leg.

**Figure 1a:**
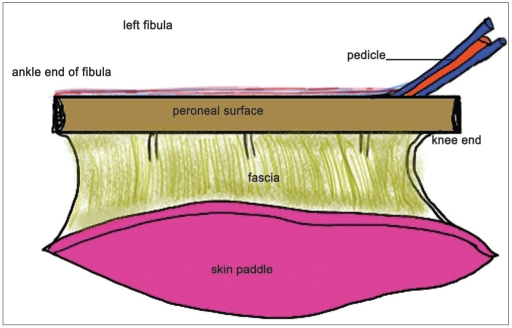
The free fibula flap from the left leg

**Figure 1b:**
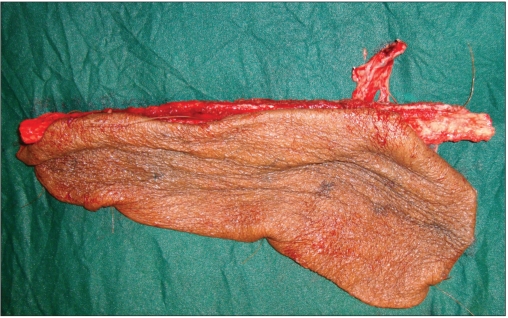
The clinical picture of Figure 1a

**Figure 2a:**
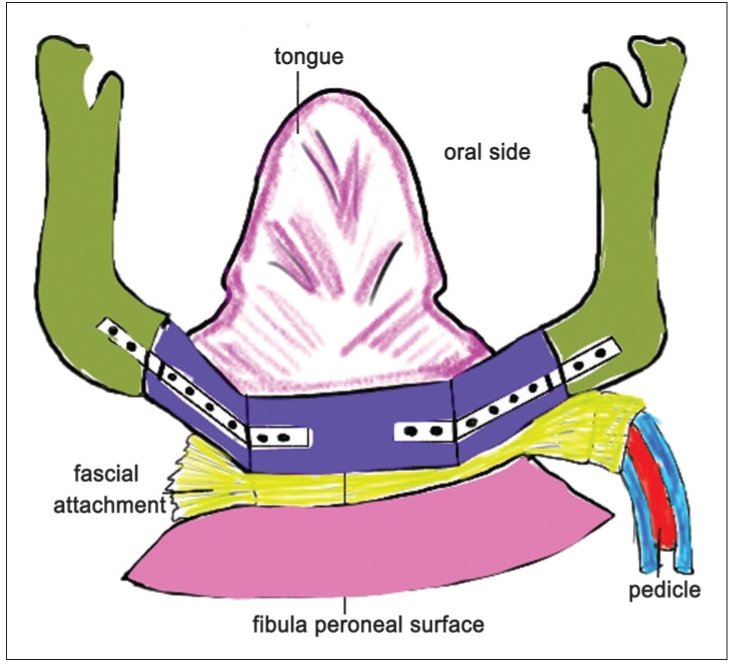
Diagrammatic representation of the left free fibula flap with left side anastomosis. The skin paddle drapes the bone from outside along with the fascia in a caudo– cranial direction as the septal attachment is in the lower border of the neomandible. The flexor hallucis longus muscle lies in the submental region

**Figure 2b:**
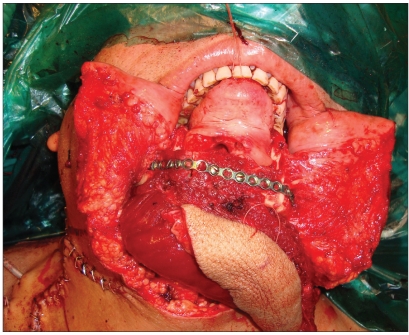
Clinical representation of Figure 2a

**Figure 3a:**
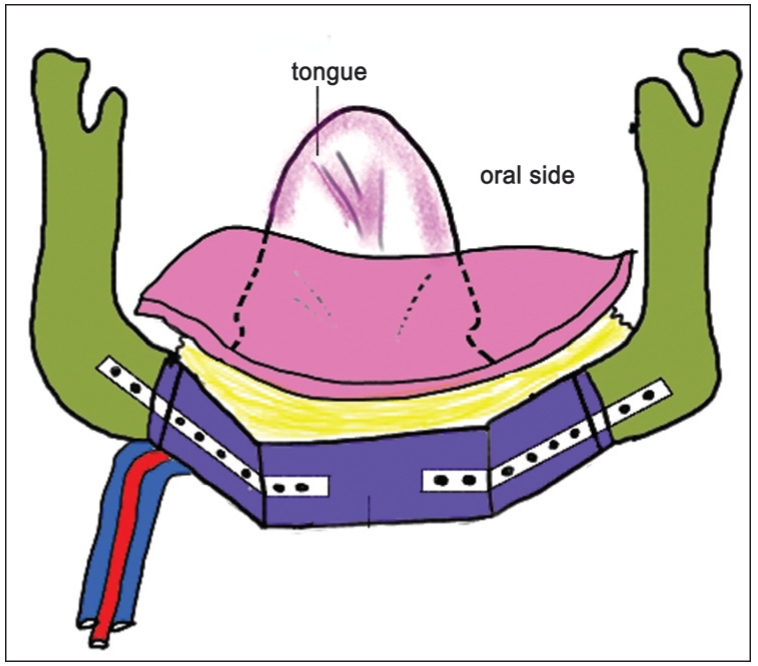
Diagrammatic representation of the left free fibula flap with right side anastomosis. The skin paddle drapes the bone from inside the oral cavity in a cranio– caudal direction as the septal attachment lies in the upper border of the neomandible. Here, the flexor hallucis longus muscle lies in the floor of the mouth

**Figure 3b:**
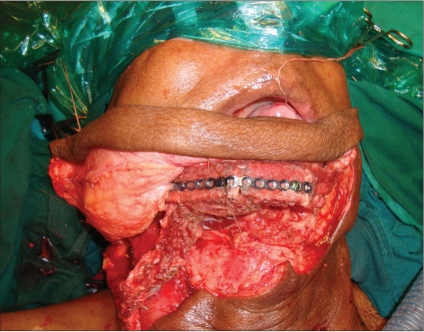
Clinical representation of Figure 3a

## RESULTS

Three hundred and thirty-four flaps survived completely. There were partial flap losses (outer paddle) in 20 patients, and superficial skin necrosis was seen in another 20. There were 39 re-explorations. Twelve flaps were lost completely. The superficial skin necrosis was managed conservatively. The lost flaps were replaced with pectoralis major myocutaneous flap, nasolabial flap, deltopectoral flap and forehead flap, either alone or in combination, depending on the requirement.

## DISCUSSION

The reconstruction of complex defects of the oromandibular region (COMD) with free flaps is a challenge because the reconstruction has to provide the missing skeletal framework, the intraoral lining and extraoral soft tissue cover. The free flap options[1,5,6] available to reconstruct the defects are fibula flap, scapula flap, radial artery flap, iliac crest, composite rib and metatarsal flaps. Owing to its distinctive advantages[1,5,6] over other flaps, the free fibula flap has become the ideal option to reconstruct COMD. The flap was first described by Taylor[7] and later popularized by Hidalgo[4–6] in the reconstruction of complex mandibular defects.

Although being the most commonly used flap in the reconstruction of COMD, there is no clear consensus available in the literature regarding the selection of the donor side. Hidalgo[3] has described that depending on the availability of the recipient vessels in the neck, the donor side can be selected. He suggested using the ipsilateral fibula for the same side defects when the recipient vessels are good. But, when the recipient vessels in the neck are not good, or are not available, and the anastomosis has to be performed on the opposite neck, he suggested to harvest the fibula contralateral to the defect side so as to facilitate the ease of anastomosis without using interposition vascular grafts. Although it is accepted universally, this may delay starting the work of the reconstructive team as the donor side has to be confirmed only after completion of the neck node dissection. Further, it requires the preparation of both the lower limbs and application of a tourniquet in both the thighs and, if the leg on the ipsilateral side has to be used, it causes crowding of the operating teams on one side, restricting the space available to the reconstructive team.

Yagi *et al*.[2] reported about the donor side selection of the fibula after considering factors such as the location of the pedicle in the neomandible, requirement of the skin paddle and orientation of the fibula to that of the remnant mandible, and presented an algorithm. But, in our study, we found that irrespective of the side of the defect and availability of neck vessels, the free fibula flap from either side can be used. Wei *et al*.[8,9] pioneered the concept of free fibula osteoseptocutaneous flap. In their study on cadavers and clinical applications, they described about the mobility and the flexibility of the posterolateral septum and the advantages of including the septum in the flap. They used the mobility of the skin paddle overlying the septum in reconstructing the complex defects of the extremity. The same concept was followed in our study in reconstructing COMD with free fibula osteosepto myocutaneous flap. By altering the design rather than the side, we were able to reconstruct defects contralateral to the side of the anastomosis without the use of vein grafts. The addition of the FHL muscle does not hinder the mobility of the septum as it is not attached to the septum and does not compress the septocutaneous perforators.

## CONCLUSION

Our study shows that the free fibula flap harvested from any leg (right or left) can be used to reconstruct COMD irrespective of the location of the defect (lateral segment or central segment or a combination of both) and availability of neck vessels. Therefore, there is no donor side specificity for free fibula in reconstructing simple or COMD.
